# Peroxides on the Surface of Organic Aerosol Particles Using Matrix-Assisted Ionization in Vacuum (MAIV) Mass Spectrometry

**DOI:** 10.1021/acs.est.3c02895

**Published:** 2023-09-11

**Authors:** Yiming Qin, Véronique Perraud, Barbara J. Finlayson-Pitts, Lisa M. Wingen

**Affiliations:** Department of Chemistry, University of California, Irvine, California 92697-2025 United States

**Keywords:** peroxides, particle surface analysis, heterogeneous oxidation, “magic” ionization, matrix-assisted ionization in vacuum (MAIV), peroxy radical self-reaction, OH oxidation

## Abstract

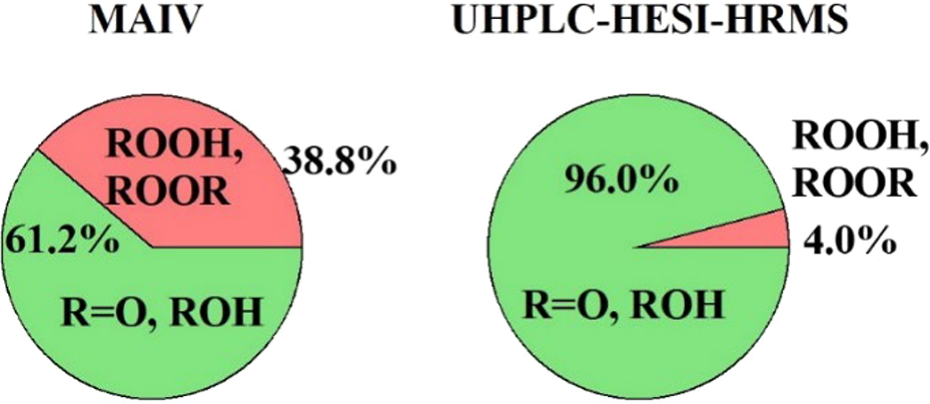

Organic peroxides are key intermediates in the atmosphere but are challenging to detect, especially in the particle phase, due to their instability, which has led to substantial gaps in the understanding of their environmental effects. We demonstrate that matrix-assisted ionization in vacuum (MAIV) mass spectrometry (MS), which does not require an ionization source, enables *in situ* characterization of peroxides and other products in the surface layers of organic particles. Hydroxyl radical oxidation of glutaric acid particles yields hydroperoxides and organic peroxides, which were detected with signals of the same order of magnitude as the major, more stable products. Product identification is supported by MS/MS analysis, peroxide standards, and offline high-resolution MS. The peroxide signals relative to the stable carbonyl and alcohol products are significantly larger using MAIV compared to those in the offline bulk analysis. This is also the case for analysis using fast, online easy ambient sonic-spray ionization mass spectrometry. These studies demonstrate the advantage of MAIV for the real-time characterization of labile peroxides in the surface layers of solid particles. The presence of peroxides on the surface may be important for surface oxidation processes as well as for the toxicity of inhaled particles.

## Abstract 2

Peroxides are efficiently detected at the surface of oxidized solid organic particles with implications for heterogeneous chemistry and health.

## 1 Introduction

Organic aerosol particles play a central role in human health, air quality, and the global climate.^1−7^ Organic aerosol particles consist of thousands of compounds with a wide range of functionalities. Previous studies mainly focused on characterizing stable compounds containing alkane, hydroxyl, carbonyl, and carboxylic acid groups. However, there is emerging evidence that reactive organic peroxides (ROOR or ROOH), produced via termination reactions of organic peroxy (RO_2_) radicals and/or HO_2_,^8−10^ can comprise a significant mass fraction of secondary organic aerosol.^11−13^ Organic peroxides are potential particle-phase oxidants that can trigger the heterogeneous oxidation of atmospheric species.^14−17^ In addition, organic peroxides are an important source of reactive oxygen species, causing oxidative stress in biological systems, including humans.^18,19^ Therefore, defining the formation and evolution of organic peroxides is essential for understanding the role of organic aerosols in atmospheric chemistry and human health.

Characterization of particle-phase organic peroxides, however, is enormously challenging due to their high lability stemming from the weakness of the peroxy O–O bond.^13^ Significant progress in organic peroxide detection has been made since the development of the iodometric spectrophotometric method.^12,20^ The iodometric method is based on the reaction between peroxide species with an iodide ion (I^–^) forming a triiodide ion (I_3_^–^) that has a characteristic UV absorption. However, this method determines the total peroxide content (including H_2_O_2_ and organic peroxides) without molecular specificity and can suffer from matrix effects.^21^ The advancement of mass spectrometry techniques, such as liquid chromatography electrospray ionization mass spectrometry (LC-ESI-MS), iodometric-LC-ESI-MS, and atmospheric-pressure chemical ionization tandem mass spectrometry (APCI-MS/MS), have also been successful in the molecular characterization of organic peroxides.^11,22−27^ These studies have established that organic peroxides are highly labile and are subject to decomposition in solvents and water or under heat on a time scale of minutes, making them difficult to detect with offline solvent extraction methods.^11,24,28^ Moreover, the reactivity and thermal stability vary significantly among different peroxides and isomeric structures,^24,26^ highlighting the potential for the underestimation of their concentrations in various systems as well as the need for real-time particle-phase organic peroxide detection.

Previous particle-phase organic peroxide detection methods gave information on the bulk composition of the particles, with no distinction between the surface and the core of the particle. Whether the organic peroxides are on the surface or in the core of the particles may impact the chemical reactivity and toxicity of the particles. Peroxides formed via the surface oxidation of highly viscous particles may remain predominantly near the gas–particle interface. Since organic peroxides have a relatively high molecular weight and O:C ratio, they are expected to diffuse slowly within highly viscous particles.^29^ Indeed, results from a reaction–diffusion kinetic model for the glutaric acid particle–OH oxidation system show that dimers from RO_2_ reactions are predicted to remain primarily near the gas–particle interface, especially in diffusion-limited viscous particles.^30^ However, direct observational evidence is lacking.

We have demonstrated previously that “magic” ionization mass spectrometry,^31−34^ so named because it does not involve an ionization source, provides molecular information regarding the composition of surface layers of organic particles in real time.^35^ The method, also known as matrix-assisted ionization in vacuum (MAIV), is based on the spontaneous emission of ions from charged particles that occurs under subatmospheric pressures at the inlet of a mass spectrometer. Particles with multiple charges undergo evaporation or sublimation when they enter the mass spectrometer due to the pressure drop. The shrinkage increases the charge repulsion and leads to the emission of ionized surface molecules to the gas phase.^35^ Since no external energy is employed, this online method minimizes the potential decomposition of the parent molecules, making it a promising approach for detecting labile compounds. In the present study, we expand the MAIV technique to the heterogeneous oxidation of glutaric acid particles by OH, in which the products observed include the ketone and the alcohol as well as peroxides. For comparison, offline bulk-particle analysis (UHPLC-HESI-HRMS) and real-time, on-the-fly easy ambient sonic-spray ionization mass spectrometry (EASI-MS) are also applied to provide information on the chemical composition of both the surface and bulk. These studies show that the signal intensities due to peroxides in the surface layers are higher than expected compared to those of previous bulk measurements. This has implications for the interactions of these peroxides present at the surface with gas-phase species and other surfaces, such as those of the respiratory system.

## 2 Methods

### 2.1 Flow Tube Experiments

#### Glutaric Acid Particle Oxidation Experiments

**Figure 1:**
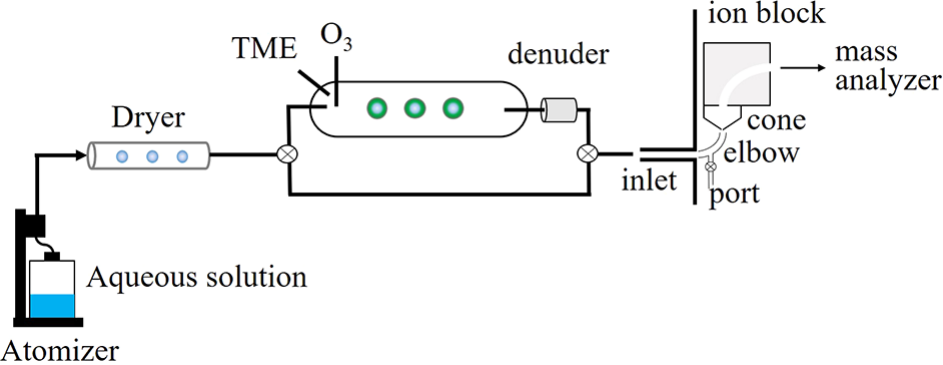
Schematic of the experimental apparatus used for the heterogeneous OH oxidation of organic particles. MAIV mass spectrometry was achieved by removing the ion source from a triple quadrupole mass spectrometer (Waters, Xevo TQ-S).

A schematic of the experimental apparatus is shown in Figure 1. A solution of 20 mM glutaric acid (Sigma-Aldrich, 99%) in 18.2 MΩ cm water (Nanopure, Millipore Corporation) was atomized at a flow rate of 3.5 L min^–1^ with purge air by using a constant output atomizer (TSI, Model 3076). Note that during the atomization process, particles experience “spray electrification” which results in the formation of charged particles.^35−39^ Atomized particles were passed through two silica gel diffusion dryers, which resulted in low relative humidity (RH < 5%). The solid particles were directed into a flow tube with a residence time of 22 s in the absence or presence of OH radicals, generated by tetramethylethylene (TME) ozonolysis.^40,41^ Ozone was generated from the passage of oxygen (Praxair, 99.993%) through a penray lamp (UV Products, Inc.) and introduced into the flow tube at 0.3 L min^–1^, giving an initial O_3_ concentration of 5 ppm in the flow tube. Tetramethylethylene (2,3-dimethyl-2-butene; Sigma-Aldrich, >99%) was injected as a liquid using a syringe pump into a flow of air (0.1 L min^–1^) that resulted in an initial concentration of 17 ppm in the flow tube. Lower or higher concentrations over the range of 4–34 ppm TME did not affect the oxidation product distribution. Typical size distributions of the particles before and after the oxidation are shown in Figure S1. For chemical analysis, the outflow from the flow tube was passed through a carbon denuder (Aerodyne Research) to remove gases. For the online methods, the particles were directly sampled with a mass spectrometer. For the offline method, particles were collected onto a Teflon filter (Fluoropore Membrane Filters, PTFE, 0.45 μm) with the aid of a pump (SKC, Inc., Leland Legacy) set at 3 L min^–1^ for 1.5 h. After the collection, the filters were immediately extracted with a 3 mL mixture of 90% Nanopure water and 10% acetonitrile (Fisher Scientific, HPLC grade) and shaken for 15 min using a Vortex Mixer (Thermolyne, Maxi Mixer II) to minimize peroxide decomposition compared to sonication.

#### Mixed Particle Oxidation Experiments

Mixtures of 20 mM glutaric acid and 0–18 mM adipic acid (Sigma-Aldrich, >99.5%) were prepared in 18.2 MΩ cm water. Internally mixed particles were generated by atomizing a single solution containing the two acids. Mixtures of glutaric acid (GA) and adipic acid (AA) solutions with molar ratios of 1:0 (pure GA) to 1:0.9 (GA:AA) were atomized and diffusion-dried before directing into the flow tube to carry out OH oxidation as for the pure glutaric acid particles. For comparison, externally mixed particles of GA and AA were produced by atomizing the 20 mM solution of pure GA and 18 mM solution of pure AA separately. The two particle streams then merged before passing through the diffusion dryers and were introduced into the flow tube.

#### Peroxide Standards

To generate particle-phase peroxides from the standards, mixtures of 20 mM GA with a commercial organic hydroperoxide or organic peroxide were also prepared, atomized, and dried. The standards included *tert*-butyl hydroperoxide (Sigma-Aldrich, 70% in H_2_O), di-*tert*-butyl peroxide (Sigma-Aldrich, 98%), and dicumyl peroxide (Sigma-Aldrich, 98%), which were readily available commercially. The molar ratios of GA to the standards were 1:1.7 for glutaric acid/*tert*-butyl hydroperoxide, 1:0.7 for glutaric acid/di-*tert*-butyl peroxide, and 1:0.3 for glutaric acid/dicumyl peroxide, which were limited by the water solubility of the compounds. Note that due to low solubility in water, the dicumyl peroxide solution was prepared in acetonitrile instead of water.

### 2.2 Mass Spectrometry

Products were identified using three approaches: (1) MAIV mass spectrometry, (2) easy ambient sonic-spray ionization mass spectrometry (EASI-MS), and (3) ultra-high-pressure liquid chromatography with high-resolution orbitrap mass spectrometry using heated electrospray ionization (UHPLC-HESI-HRMS).

#### MAIV

MAIV mass spectrometry was achieved by removing the ion source of a triple quadrupole mass spectrometer (Waters, Xevo TQ-S), as described previously.^35^ In these experiments, glutaric acid is the particle-phase reactant and behaves as a matrix that carries species from the surface into the gas phase. The mechanism of ion ejection from the surface of a solid particle into the gas phase is not clear. Particles acquire charges during atomization through “spray electrification”.^35−39^ When the charged particles enter the mass spectrometer, the diameter decreases due to the sublimation under subatmospheric pressure. By analogy to the charged residue mechanism (CRM) and the ion evaporation mechanism (IEM), the concentration of the charges on the decreasing surface area leads to the ejection of ions into the gas phase. During ion ejection, molecules that coexist in the surface layers are also ejected as ions, leading to the surface sensitivity of the method. In our previous study,^35^ the ion signals detected as the shrinking occurred were reasonably well-matched by both the CRM and IEM models. However, CRM and IEM are for liquid water droplets, and research to understand the specific mechanism occurring in solids is underway using computational methods.

The source parameters were as follows: the temperature-controlled ion block (“source temperature”) was operated at 150 °C unless otherwise noted, cone voltage 30 V, and source offset 50 V. The source temperature is applied to the instrument ion block but the temperature experienced by the particles as they travel through the inlet elbow and cone (Figure 1) is lower, as discussed in our previous work.^35^ The applied source temperature of 150 °C corresponds to 61 °C at the elbow, as measured with an infrared thermometer (Etekcity, Lasergrip 774). Both positive and negative ion mode mass spectra were collected in a continuum mode from 20–500 amu. Where noted, multichannel analysis (MCA) mode scans were collected to increase signal-to-noise ratios. MS/MS spectra were collected with MCA mode for 10–30 min. For these, the entrance and exit voltages of the collision cell were set to 1.0 (arbitrary units), with a collision cell pressure of 3 × 10^–3^ mbar and an Ar collision gas flow rate of 0.09 mL min^–1^.

#### EASI-MS

The triple quadrupole mass spectrometer was interfaced to a custom-built nebulizer described in detail elsewhere.^42^ Briefly, there are two modes of operation using EASI-MS. The EASI-orthogonal mode, with the particle stream located at a 90° angle and 10 cm from the output beam of solvent droplets from the nebulizer, probes the molecules in the surface layers of the particle. In the EASI-droplet mode, the particle stream intersects the solvent droplets close to the nebulizer exit, which leads to the uptake and dissolution of the particles and hence a measure of the bulk composition. The nebulizing solution was 50% Nanopure H_2_O and 50% methanol (Sigma-Aldrich, HPLC grade, ≥99.9%) with 0.1% formic acid (Sigma-Aldrich, >95%). The source parameters on the mass spectrometer during EASI-MS measurements were the same as for MAIV. Negative ion mode mass spectra were collected in the continuum mode from 20–500 amu.

#### UHPLC-HESI-HRMS

Analysis by UHPLC-HESI-HRMS provided product separation and accurate mass measurements using a Q Exactive Plus Orbitrap high-resolution mass spectrometer coupled to a Vanquish Horizon UHPLC system (Thermo Scientific). The UHPLC system allowed for the separation of products, and the molecular formula of each product was obtained from high-resolution MS data. Full scans in ESI (−) ion mode with the range 100–500 amu were used. The detailed parameters of UHPLC-HESI-HRMS can be found in Text S1.

## 3 Results and Discussion

### 3.1 Detection of Organic Peroxides from Glutaric Acid Oxidation

**Figure 2:**
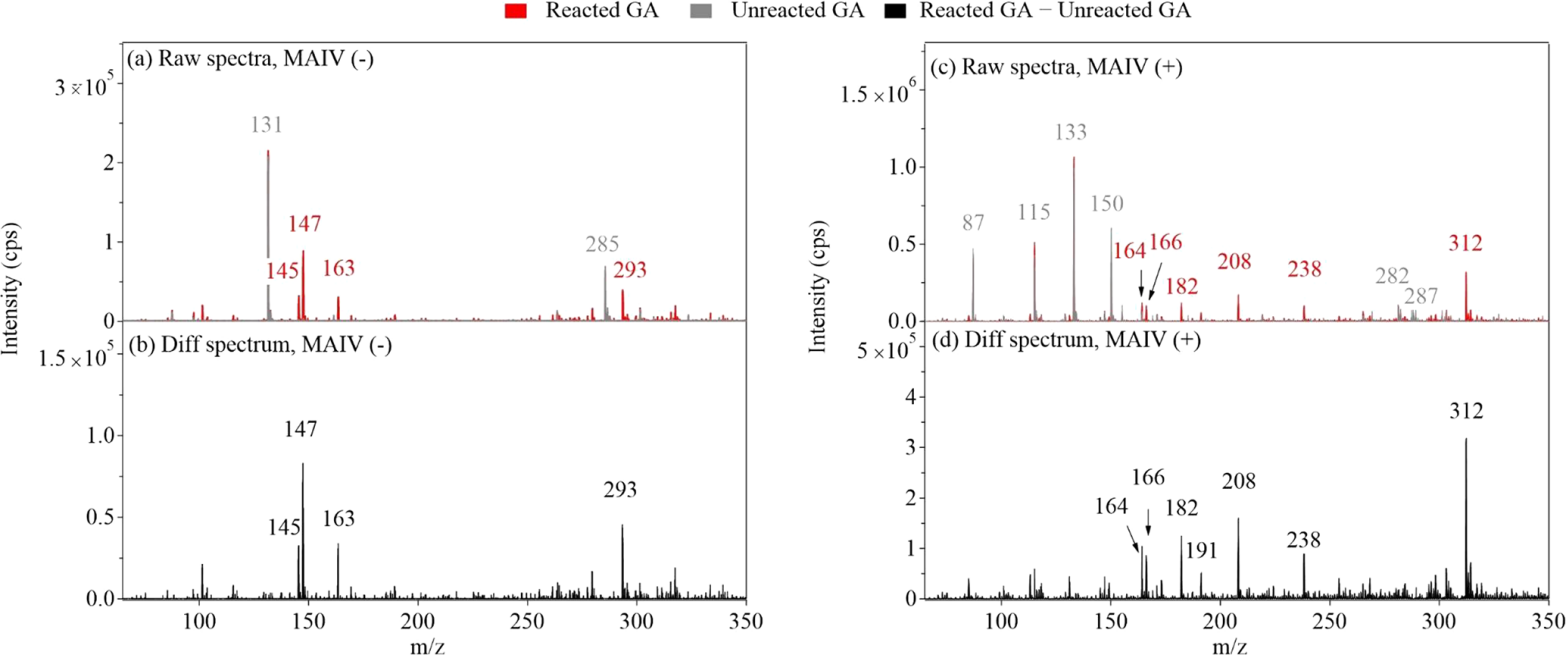
Mass spectra of glutaric acid before and after OH oxidation using surface-sensitive MAIV mass spectrometry in both negative (−) and positive (+) ion modes. Unreacted glutaric acid particle spectra are gray, reacted spectra are red, and difference spectra are black. Peak assignment in negative ion mode: [GA – H]^−^, *m*/*z* 131; [(HOOC(CH_2_)_3_COO)_2_Na]^−^, *m*/*z* 285; [(R=O) – H]^−^, *m*/*z* 145; [ROH – H]^−^, *m*/*z* 147; [ROOH – H]^−^, *m*/*z* 163; [ROOR – H]^−^, *m*/*z* 293. Peak assignment in positive ion mode: [GA + H]^+^, *m*/*z* 133; [M + NH_4_]^+^, *m*/*z* 150; [M + Na]^+^, *m*/*z* 155; [2M + NH_4_]^+^, *m*/*z* 282; [2M + Na]^+^, *m*/*z* 287; [GA + H – H_2_O]^+^, *m*/*z* 115; [GA + H – H_2_O – CO]^+^, *m*/*z* 87; [(R=O) + NH_4_]^+^, *m*/*z* 164; [ROH + NH_4_]^+^, *m*/*z* 166; [ROOH + NH_4_]^+^, *m*/*z* 182; [ROOR + NH_4_]^+^, *m*/*z* 312; [C_7_H_10_O_6_ + H]^+^, *m*/*z* 191; [C_7_H_10_O_6_ + NH_4_]^+^, *m*/*z* 208; [ROOR′ + NH_4_]^+^, *m*/*z* 238. The R denotes GA, and the R′ denotes the CH_3_C(O)CH_2_ of acetone.

**Figure 3:**
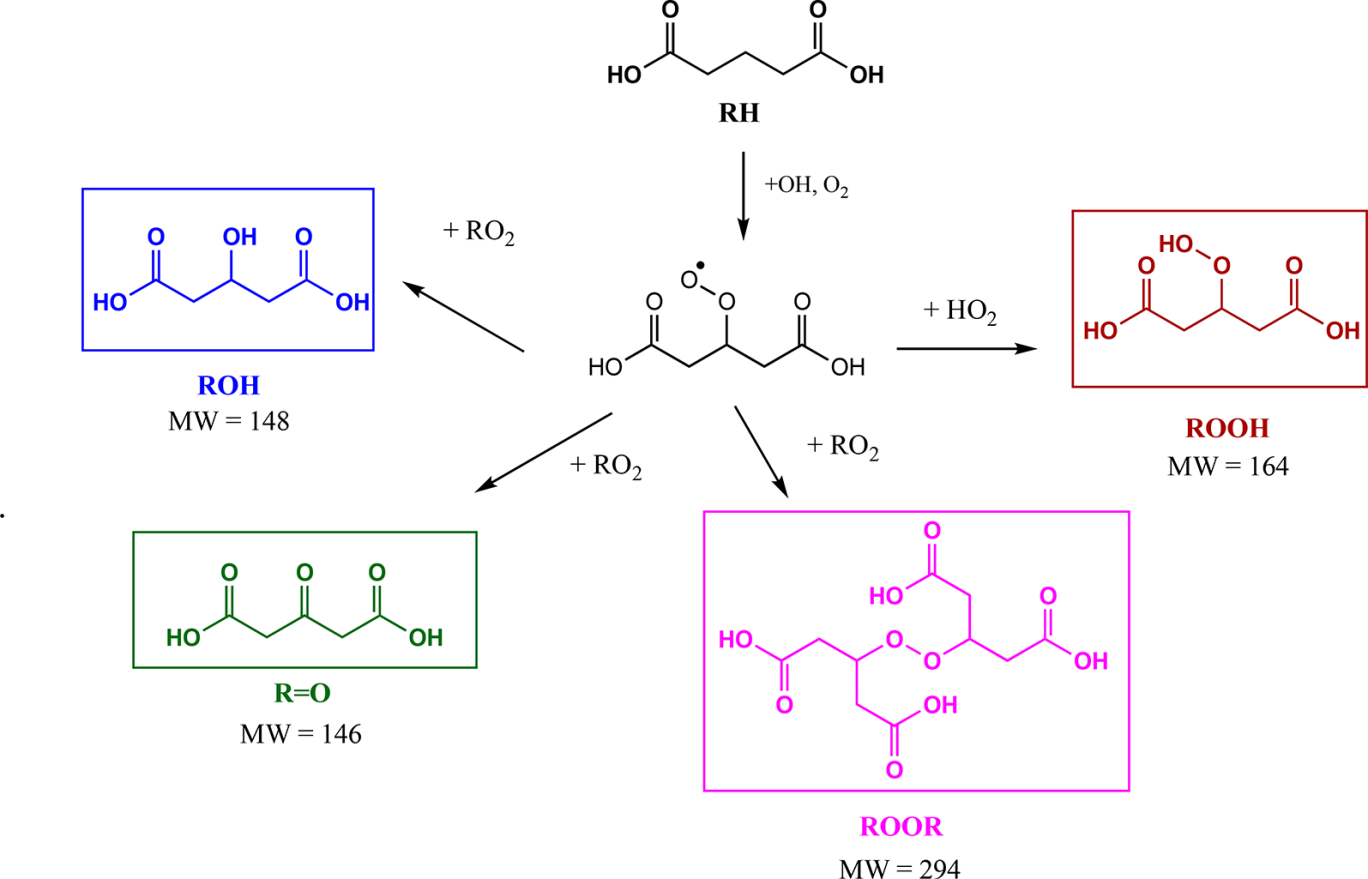
Reaction mechanism for glutaric acid OH oxidation based on well-known chemistry.^43^ Products shown are for the hydrogen abstraction from the central carbon (C_β_), which is the most reactive.^46^

Figure 2 shows the spectra of glutaric acid (GA) before and after OH oxidation using surface-sensitive MAIV mass spectrometry. The raw spectra from the negative ion mode with reacted GA and unreacted GA are shown in Figure 2a, while the difference between the two spectra is shown in Figure 2b. In the unreacted spectrum (2a, gray), the base peak is at a mass-to-charge ratio (*m*/*z*) of 131, corresponding to [M – H]^−^, the parent ion of GA (MW 132). After oxidation, peaks at *m*/*z* 145, 147, 163, and 293 were observed. The mechanism of OH oxidation for GA is shown in Figure 3. Based on well-known chemistry,^43^ OH radicals abstract a hydrogen atom from glutaric acid, and O_2_ adds to form a peroxy radical (RO_2_). In the absence of NO_*x*_, RO_2_ radicals react with other RO_2_ or with HO_2_:^43^
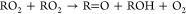
I
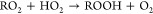
II
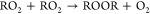
III

The more stable ketone and alcohol products from channel I have been identified as the main products.^13,30,44−46^ Reaction channels II and III, which form the organic peroxides, have been considered to be minor pathways, but this may be in part due to the difficulty in detecting them.^13,47−49^ For example, in the study by Zhao et al.^30^ on glutaric acid particle oxidation at the gas–particle interface,^30^ organic peroxides formed from channels II and III were detected in low intensities (less than 4% in terms of total signal intensity). However, branching ratios for ROOR formed in the gas phase ranging from 10%^50^ up to 23%^51^ have recently been reported.

In the positive ion mode (Figures 2c and 2d), major peaks for unreacted GA include [M + H]^+^ (*m*/*z* 133), [M + NH_4_]^+^ (*m*/*z* 150), [M + Na]^+^ (*m*/*z* 155), [2M + NH_4_]^+^ (*m*/*z* 282), and [2M + Na]^+^ (*m*/*z* 287), as well as smaller fragments corresponding to [M + H – H_2_O]^+^ and [M + H – H_2_O – CO]^+^ at *m*/*z* 115 and 87, respectively. The signals from *m*/*z* 164, 166, 182, and 312 are observed after oxidation, corresponding to the ammoniated adducts of R=O, ROH, ROOH, and ROOR, respectively. Due to the presence of ammonia in ambient air, ammonium adducts are commonly observed in ambient ionization techniques and are especially common for polar compounds.^52^ Small sodiated adducts are present from trace metals in the glassware.^53^ Peaks at *m*/*z* 191, 208, and 238 were also observed in the positive ion mode after oxidation. The *m*/*z* values of 191 and 208 can be related to a glutaric acid alkoxy radical (RO) decomposition product (see Text S2). The peak at *m*/*z* 238 is attributed to an ROOR′ product formed between the glutaric acid RO_2_ radical and an acetone R′O_2_ radical formed from TME ozonolysis (see Text S2). Additional products, for example, RO_2_ autoxidation products or esters, as suggested in previous studies,^30,54^ were not observed. These reaction pathways may be inhibited in our case, possibly due to the dissipation of energy and steric hindrance in the solid glutaric acid particles. Second-generation products from the OH attack were also not observed due to the short residence time of the particles in the flow tube.

Product identification was further carried out with MS/MS at different collision energies (CE) (Figures S2–S5 and Text S3) and is consistent with the proposed products. Specifically, the fragmentation of the hydroperoxide [M + NH_4_]^+^ adduct (Figure S4) proceeds via the loss of neutral NH_3_, leading to protonated molecules consistent with the molecular weight of the ROOH product. It then loses one H_2_O to yield the RO^+^ fragment (*m*/*z* 147) which is common for peroxides.^55^ Further loss of a neutral CH_3_COOH and CO_2_ results in smaller fragments at *m*/*z* 87 and 43. For the ROOR [M + NH_4_]^+^ adduct (Figure S5), neutral NH_3_ is also lost first, followed by the loss of two H_2_O molecules to yield fragments *m*/*z* 277 and 259 and the loss of CO (*m*/*z* 231), which leaves a fragment with three remaining carbonyl groups and a peroxide bond. At a higher collision energy, fragmentation occurs at the O–O bond, leading to an RO^+^ fragment (*m*/*z* 147), followed by losses of either O (*m*/*z* 131), H_2_O (*m*/*z* 113), and CO (*m*/*z* 85) or O (*m*/*z* 131), CO_2_ (*m*/*z* 87), and CO_2_ (*m*/*z* 43).

To further confirm that MAIV detects peroxides, MAIV spectra of several commercially available organic peroxide standards mixed with glutaric acid as the matrix were also collected (as shown in Figure S6). For all peroxide standards, the source temperature of the mass spectrometer was kept at 30 °C to minimize thermal decomposition. The parent ions [ROOR + NH_4_]^+^ or [ROOH + NH_4_]^+^ as well as the RO^+^ fragment were observed in all of the standards; the latter is consistent with the breaking of the O–O bond in the glutaric acid ROOH and ROOR products under collisional dissociation. Further experiments were conducted for the dicumyl peroxide and glutaric acid mixture as a function of the source temperature (Figure S7). An increase in the source temperature increases the signal intensity of GA and slightly increases the intensity of dicumyl peroxide (Figure S7a). The increase in signal intensity is likely due to the enhanced sublimation of glutaric acid and higher ionization efficiency of the molecules at higher temperatures.^35^ The relatively smaller increase in the dicumyl peroxide parent [M + NH_4_]^+^ peak results from a combination of the higher ionization efficiency and greater decomposition to RO^+^ at higher temperatures. Indeed, at source temperatures greater than 70 °C, corresponding to 39 °C at the elbow,^35^ the relative signal of the ROOR parent peak decreased while the fragment peak increased (Figure S7b). In the case of peroxides from glutaric acid oxidation, the decomposition product (RO^+^) was not observed (Figures 2c and 2d). Therefore, the high source temperature was suitable for the glutaric acid oxidation experiment to maximize ionization efficiency. The results herein confirm the ability to detect organic peroxides using MAIV.

Additional molecular identification was carried out by collecting both unreacted and OH-reacted particles onto a Teflon filter to conduct offline UHPLC-HESI-HRMS. Shown in Figure S8 are extracted ion chromatograms (EIC) of the GA reactant and product peaks observed in the negative ion mode at *m*/*z* 131 [GA – H]^−^, *m*/*z* 145 [(R=O) – H]^−^, *m*/*z* 147 [ROH – H]^−^, *m*/*z* 163 [ROOH – H]^−^, and *m*/*z* 293 [ROOR – H]^−^. The product peaks only appear in the oxidation sample but not in the unreacted sample, eliminating possible artifacts or contamination during sample preparation. Table S1 lists the compound retention time, observed accurate mass, exact mass, assigned formula, mass accuracy, and peak area. The accurate mass and exact mass of each ion are within ±1 ppm, further supporting the product identification.

### 3.2 Relative Ratio of Organic Peroxides from Glutaric Acid Oxidation

**Figure 4:**
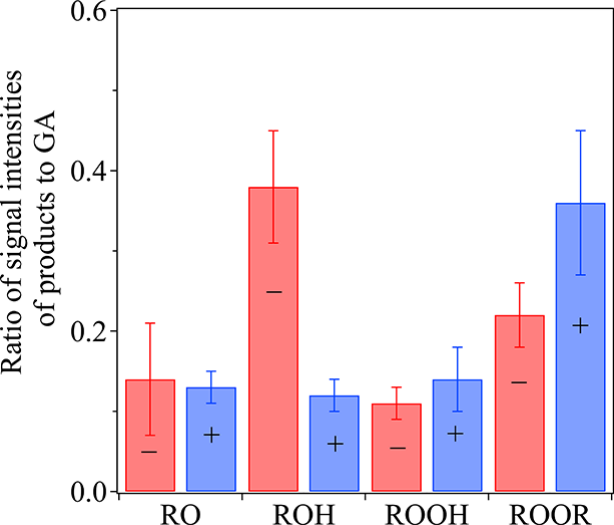
Relative ratios of signal intensities for products compared to glutaric acid from MAIV mass spectrometry in both positive ion (blue) and negative ion (red) modes. The averages and the standard deviations were obtained from an average of five mass spectra with the same experimental conditions but conducted on different days.

While MAIV is known to be sensitive to surface species, the actual probing depth is yet unknown, making quantification challenging. Therefore, the product intensities in both the positive and negative ion modes were ratioed to the reactant GA signals for comparison (Figure 4). The relative signals for ROOH and ROOR to GA are on the same order of magnitude as the more stable products, regardless of the ion mode used. While absolute concentrations could not be derived, the signal intensity ratios for the peroxide products are similar to those for the alcohol and ketone products, indicating larger contributions of peroxides to heterogeneous product formation than those previously reported. These higher signal intensities reflect the gentle nature of MAIV and the fast online analysis, which minimize the decomposition of peroxides. As noted above, gas-phase yields of ROOR have also been recently reported to be higher than expected;^50,51^ thus, this chemistry is not specific to the particle surface.

**Figure 5:**
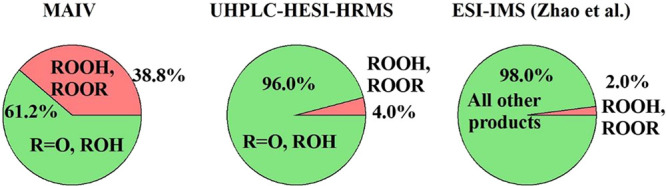
Relative signal intensities for MAIV (−), UHPLC-HESI-HRMS (−), and ESI-IMS (−) from glutaric acid oxidation by OH radicals. The proportion of peroxides from MAIV analysis is 39 ± 11% (1s) of the product intensity. The ESI-IMS result was reported by Zhao et al.^30^

Figure 5 further compares the product distributions obtained from MAIV and those from UHPLC-HESI-HRMS to a literature-reported distribution using ESI ion mobility mass spectrometry (ESI-IMS).^30^ All of the results were obtained from negative ion mode peak intensities for consistency. The labile products, including ROOR and ROOH, show much higher proportions (39 ± 11% (1s)) with MAIV as compared to the 4% in the UHPLC-HESI-HRMS method, which is similar to the 2% reported by Zhao et al.^30^ The comparison of MAIV with UHPLC-HESI-HRMS demonstrates the significant advantage of MAIV for the real-time characterization of labile peroxide compounds.

### 3.3 Enhanced Detection of Surface-Bound Oxidation Products

**Figure 6:**
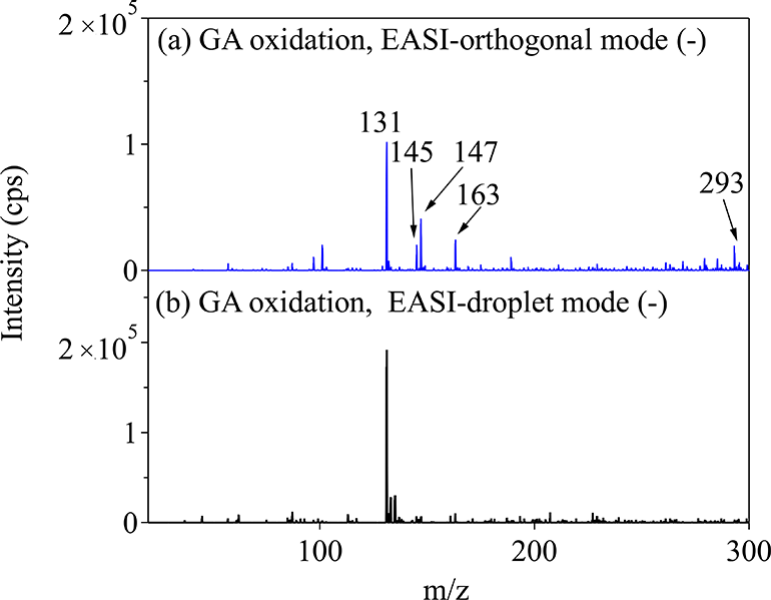
EASI (−) mass spectra for glutaric acid particles after OH oxidation acquired in (a) the surface-sensitive orthogonal mode and (b) the bulk-sensitive droplet mode.

To compare the surface vs the bulk composition, the reacted particles were further analyzed with EASI-MS, which is a real-time ambient ionization solvent-spray technique with no applied voltage.^42,56^ The probing of the surface vs the bulk is achieved by changing the configurations and the distance between the particle flow and the charged solvent flow from the nebulizer.^42^ Shown in Figure 6 are the EASI (−) spectra, with product peaks at *m*/*z* 131, 145, 147, 163, and 293 in the EASI-orthogonal mode that probes the surface and the EASI-droplet mode that probes the bulk. The product intensities were ratioed to the reactant GA signals (Figure S9). The relative signals for each product to GA in the orthogonal mode are similar to those for MAIV, consistent with all products being present in the surface layers. For the droplet mode, in which the whole particle is dissolved, the product ratios to GA are lower, consistent with this being a bulk-particle method. Additionally, all products are about the same order of magnitude for detection by each method, indicating that the labile products make up a significant fraction of the total products for the EASI online methods, which is a faster and softer method. The spectra from the surface resemble those from MAIV, while the spectra from the bulk predominantly show a signal from the unreacted glutaric acid core. For comparison, the experiments with the unreacted GA in the EASI-orthogonal and EASI-droplet modes are shown in Figure S10.

The results from these surface and bulk measurements using EASI-MS support the idea that the products, including the organic peroxides, are confined to the particle surface. Although EASI-MS analysis also involves solvent extraction during the interaction of the particle beam with the output of the nebulizer, the particles are in contact with the solvent for a very short time, on the order of milliseconds. It is this fast online sampling approach of EASI-MS and MAIV that allows these labile species to be detected. This is in contrast to offline UHPLC-HESI-HRMS analysis, in which the particles spend several minutes in the solvent during separation and analysis and are exposed to high temperatures during the heated electrospray process. Although the formation of organic peroxides was observed as a minor contribution in past studies, the results here show this is an important reaction pathway for the oxidation of organic particles, especially at the particle surface.

**Figure 7:**
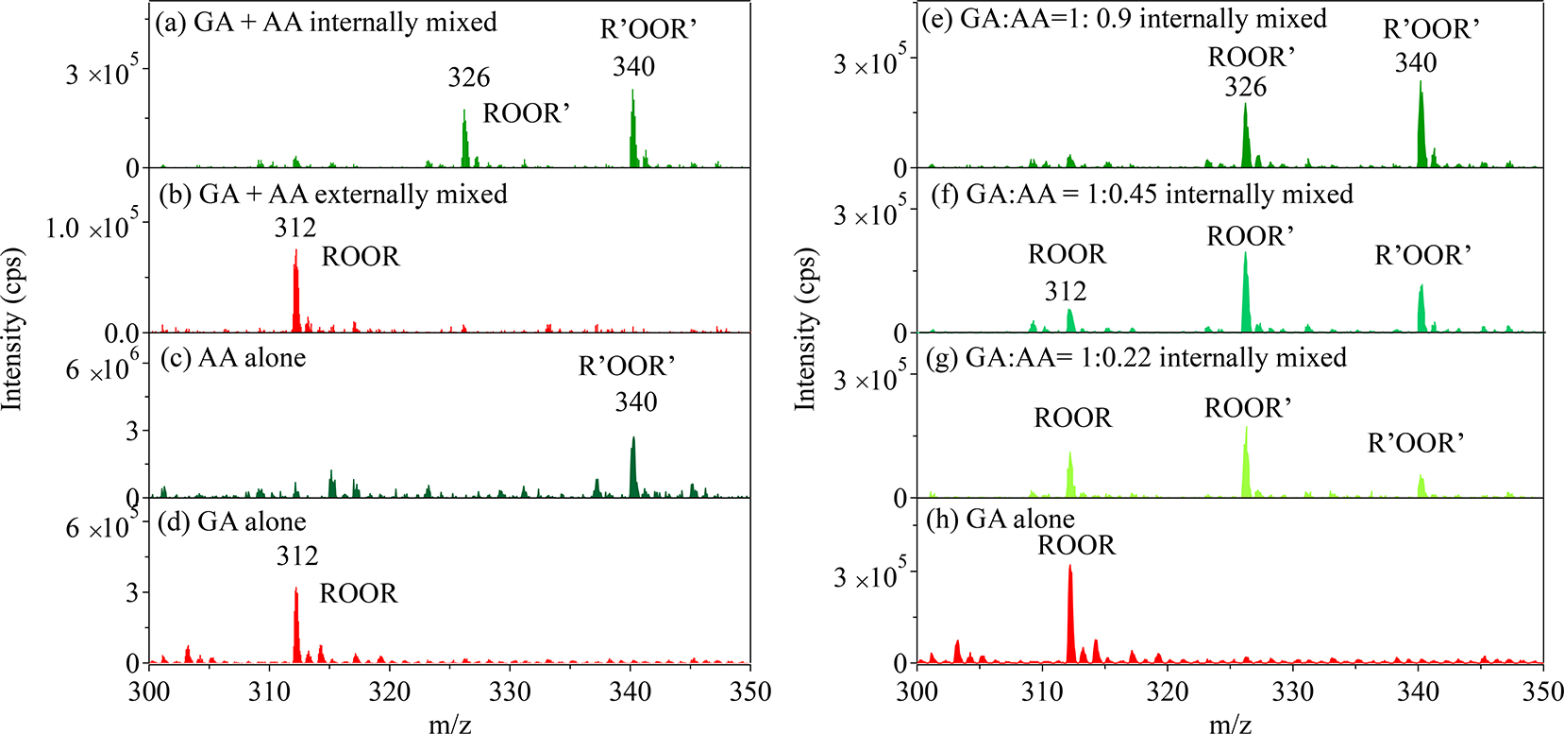
MAIV (+) spectra of peroxide products from pure diacids (ROOR or R′OOR′) or the cross-product (ROOR′) obtained from various mixtures of reactants. (a) Internally mixed glutaric acid and adipic acid particles, (b) externally mixed glutaric acid and adipic acid particles, (c) pure adipic acid particles, and (d) pure glutaric acid particles. (e–h) Internally mixed glutaric acid and adipic acid particles with molar ratios of (e) 1:0.9, (f) 1:0.45, and (g) 1:0.22 and (h) glutaric acid particles alone. Note that the spectrum in (c) was collected using an additive MCA scan of 5 min while other spectra were collected using the averages of continuum scans. The use of MCA was due to the lower ionization efficiency of atomized adipic acid particles.

To further explore how the ROOR forms on the surface, a search for a cross ROOR′ product was carried out using both internally mixed and externally mixed glutaric acid (R) and adipic acid (R′) particles (Figure 7). The [ROOR + NH_4_]^+^ product is observed at *m*/*z* 312 for pure glutaric acid particles (Figure 7d), while the corresponding [R′OOR′ + NH_4_]^+^ product is observed at *m*/*z* 340 for pure adipic acid particles (Figure 7c). The cross-product consistent with ROOR′ (*m*/*z* 326) formed from glutaric acid RO_2_ and adipic acid R′O_2_ is only observed in the internally mixed particles (Figures 7a and 7b). Given the lower ionization efficiency of adipic acid, the R′OOR′ product in the externally mixed particles (Figure 7b) is likely below the detection limit.^35^ Shown in Figures 7e–7h are the internally mixed glutaric acid and adipic acid particles with a series of different molar ratios of GA to AA. A gradual shift to ROOR′ and then R′OOR′ is evident with increasing amounts of AA in the particles. The results here suggest that peroxide formation occurs from the self-reaction of alkylperoxy radicals that are co-located on the same particle surface.

Several factors contribute to surface-bound oxidation products. It has been well-established that the reactions of OH with high-viscosity semisolids occur primarily in the surface layer, giving a highly oxidized surface crust.^30,57−61^ The surface concentrations of alkylperoxy radicals formed from hydrogen abstraction followed by the addition of O_2_ can thus be enhanced relative to the gas phase, resulting in increased self-reactions. Changes in reactivity as the chemistry becomes confined to an interface have been simulated in previous studies.^57^ In the case of the OH–glutaric acid reaction, the formation of peroxides along with other products from RO_2_ + RO_2_ reactions at low relative humidity has been reported, and modeling studies predicted that they are confined to the top few nanometers of the particles.^30^ Further studies are warranted for the detection of surface species in other heterogeneous oxidation systems.

## 4 Atmospheric Implications

This study demonstrates the advantage of MAIV for the real-time, on-the-fly characterization of oxidation product formation in the surface layers of solid particles with specific benefits for the sensitive detection of peroxides. To the best of our knowledge, MAIV of the oxidized surface of glutaric acid particles provides the first analytical measurement of peroxides formed at and confined to the interface of solid particles. Additionally, it highlights the importance of surface-sensitive analytical techniques for understanding the heterogeneity of aerosol particles. The surface-sensitive methods show much larger peroxide signals than expected relative to the bulk methods, supporting a significant reaction pathway for RO_2_ radicals at the gas–particle interface. The presence of peroxides at the interface can have significant implications for both heterogeneous chemistry and the toxicity of aerosol particles. For example, the formation of peroxides at the surface of particles would allow direct interaction within the respiratory system upon inhalation, relative to the case in which peroxides are buried in the bulk of the particles. The uptake of water vapor, either from the atmosphere or within the respiratory system, onto particles containing surface-bound peroxides may lead to reactive oxygen species at the particle surface through their decomposition.^18,62,63^ On the surface, the peroxides may lead to the oxidation of trace atmospheric species such as SO_2_ and aldehydes.^14,15,17^ Moreover, the deposited particles on indoor surfaces could play a significant role in the heterogeneous oxidation of adsorbed organics if peroxides are present at the particle interface. The molecular identification of dimers and other species at the surface of particles can also contribute to the understanding of physical aerosol properties, such as aerosol viscosity and volatility, that are important for developing predictive capabilities for the impacts of particles on visibility, climate, and health.

## Supporting Information Available

Additional experimental details and results of offline chromatographic analysis, particle size distributions for unreacted and OH-reacted glutaric acid particles, details of additional oxidation products, MAIV (+) MS/MS spectra of oxidation products, MAIV (+) mass spectra and source temperature dependence of peroxide standards, comparison of product intensity ratios to glutaric acid for the EASI-MS (−) droplet and orthogonal modes, and EASI (−) mass spectra of unreacted glutaric acid particles (PDF)
