# Supplementary material for: Peroxides on the Surface of Organic Aerosol Particles Using Matrix-Assisted Ionization in Vacuum (MAIV) Mass Spectrometry
Source: Environ Sci Technol. 2023 Sep 11;57(38):14260–8. doi: 10.1021/acs.est.3c02895 (PMC10537442; doi:10.1021/acs.est.3c02895)
Supplement: Supplementary file 1 — es3c02895_si_001.pdf [file es3c02895_si_001.pdf]

Supporting Information

for

Peroxides on the Surface of Organic Aerosol Particles Using Matrix Assisted Ionization in Vacuum (MAIV) Mass Spectrometry

Yiming Qin, Véronique Perraud, Barbara J. Finlayson-Pitts and Lisa M. Wingen*

Department of Chemistry, University of California, Irvine, CA, 92697-2025

* Corresponding author: Email wingenit@uci.edu; phone (949) 824-2530; FAX (949) 824-2420.

Environmental Science & Technology

Number of pages: 19
Number of figures: 10
Number of tables: 1

Summary:
Figure S1: Typical size distributions of unreacted GA particles and OH-reacted GA particles; **Text S1**: Detailed parameters used in UHPLC-HESI-HRMS; **Text S2**: Description of additional oxidation product formation; **Text S3**: Description of MAIV (+) MS/MS spectra of oxidation products; **Figure S2**: MAIV (+) MS/MS spectrum of the ketone product; **Figure S3**: MAIV (+) MS/MS spectrum of the alcohol product; **Figure S4**: MAIV (+) MS/MS spectrum of the hydroperoxide product; **Figure S5**: MAIV (+) MS/MS spectrum of the peroxide product; **Figure S6**: MAIV (+) mass spectra of commercially available peroxide standards mixed with glutaric acid; **Figure S7**: Source temperature dependence of MAIV (+) signal intensities of glutaric acid and dicumyl peroxide standard; **Figure S8**: UHPLC-HESI-HRMS (-) extracted ion chromatograms of unreacted and OH-reacted GA particles; **Table S1**: Retention times, accurate masses, exact masses, assigned formulae, mass accuracy, and peak area for each product observed using UHPLC-HESI-HRMS (-); **Figure S9**: Ratios of product signal intensities relative to glutaric acid signal for EASI-MS (-) droplet and orthogonal modes; **Figure S10**: EASI (-) mass spectra in orthogonal and droplet mode for unreacted glutaric acid particles.

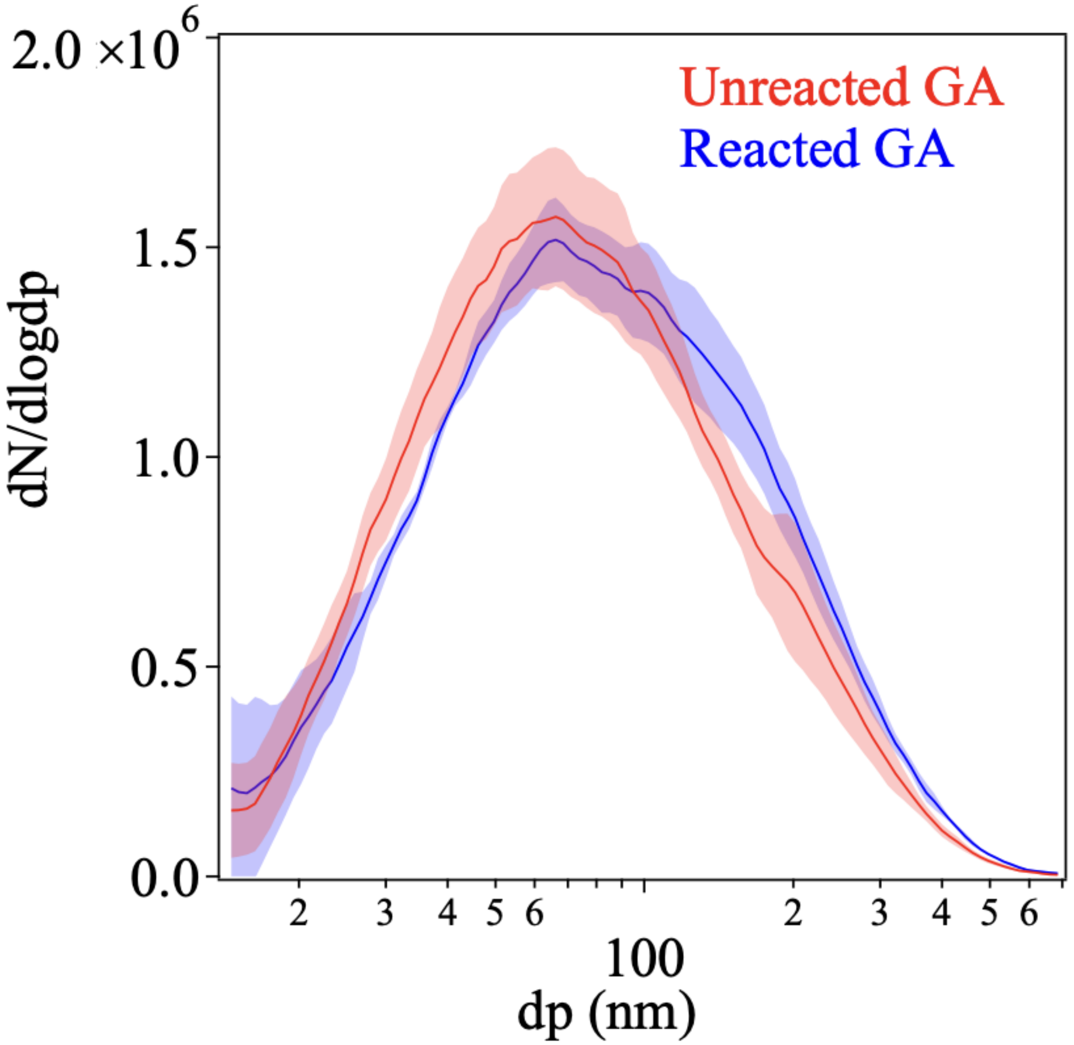

Figure S1. Typical size distributions of the unreacted GA particles and OH-reacted GA particles.

Text S1:

10 µL of the sample was injected onto a Luna Omega 1.6 µm Polar C18 150 × 2.1 mm column (Phenomenex) equipped with a SecurityGuard ULTRA cartridge (porous polar C18, 2.1 mm; Phenomenex), both maintained at 30 °C. The mobile phase flowing at 300 µL min^{-1} was composed of two solvents: solvent A: H_2O (Fisher Chemical, Optima, LCMS grade) containing 0.1% formic acid (Fisher Chemical, Optima, LCMS grade), and solvent B: acetonitrile (Fisher Chemical, Optima, LCMS grade) containing 0.1% formic acid. A gradient elution technique was applied, with a 3-minute hold at 5% B, followed by a linear increase to 95% B over a 14-minute period, holding at 95% B for 2 minutes, and finally, a return to 5% B at the end (6 minutes). A heated electrospray source (HESI; Thermo Scientific) was utilized with a capillary voltage set to 4.0 kV, a capillary temperature maintained at 325°C, a sheath gas flow rate of 35 (arbitrary units; a.u.), an auxiliary gas flow rate of 10 (a. u.), a sweep gas flow rate of 8 (a. u.), an S-lens RF level at 30 (a. u.), and an auxiliary gas heater temperature of 300°C.

The mass spectrometer was calibrated weekly for mass accuracy using the Thermo ESI (+) and ESI (-) Pierce™ LTQ Velos calibration solutions (Thermo Scientific) along with a 5 mM sodium formate calibration solution covering the range of 90 to 800 amu. Data were analyzed using Thermo Scientific FreeStyle™ 1.6 and the mass tolerance for all identified formulas was within 3 ppm.

Text S2:

The additional peaks at *m/z* 208 and 238 (Fig. 2) in the MAIV (+) MS spectra are likely to be ammoniated adducts of compounds with MW 190 and 220, respectively. A peak at m/z 191 corresponding to the $[M + H]^+$ parent of the product with MW 190 was also observed in MAIV

as well as in UHPLC-HESI-HRMS with a neutral elemental formula of $C_7H_{10}O_6$ at a retention time of 6.75 min. This product may be formed from RO radical decomposition. We propose the formation of methane triacetic acid (3-(carboxymethyl)pentanedioic acid) through the following mechanism in which RO radicals undergo decomposition and react further with neighboring glutaric acid alkyl radicals:

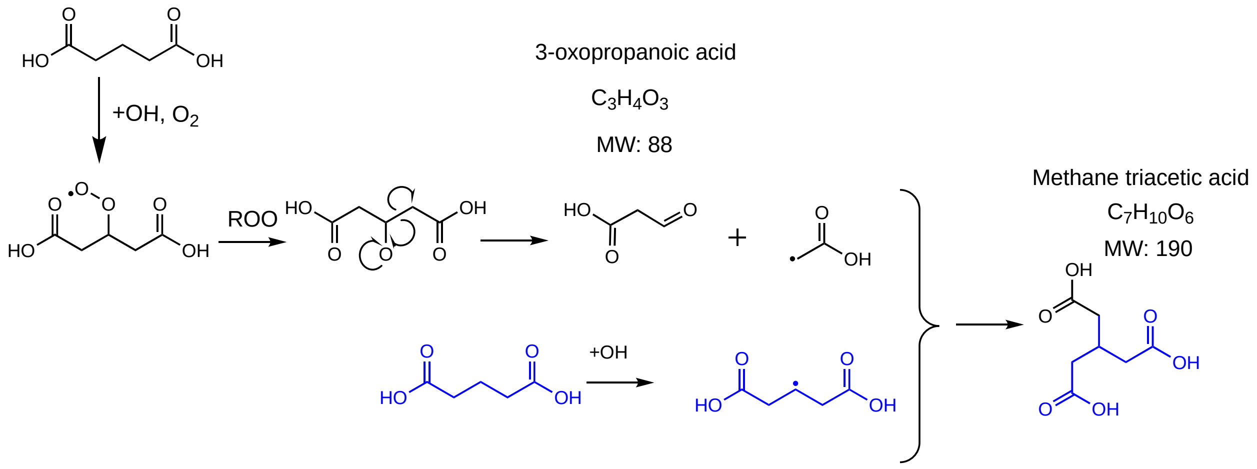

Evidence for similar decomposition of alkoxy radicals has been reported to occur during the reaction of OH with solid triacontane ($C_{30}H_{62}$) at short reacto-diffusion lengths,[1] such as those occurring for OH reactions with high viscosity particles and solid organic films.[2–6] The aldehyde co-product of the alkoxy radical decomposition, 3-oxopropanoic acid, was not observed but may have volatilized from the particles given its low enthalpy of vaporization (52 kJ mol^{-1})[7] relative to that of glutaric acid (130 kJ mol^{-1})[8]. The particle size distribution did not decrease after reaction (Fig. S1) and in fact increased slightly, suggesting that the majority of the products remained in the particle phase.

The product with MW 220 is assigned to an ROOR' product formed between the glutaric acid RO_2 radical and acetone $R'O_2$ radical produced during ozonolysis of TME. Acetone $R'O_2$ radical can be formed from the Criegee intermediate from TME ozonolysis as well as the from attack of OH on acetone and the addition of O_2.[9] The resulting $\cdot OOCH_2C(O)CH_3$ radical, which

can react with surface-bound glutaric acid RO_2, leads to the formation of an ROOR' with MW 220 ($C_8H_{12}O_7$).

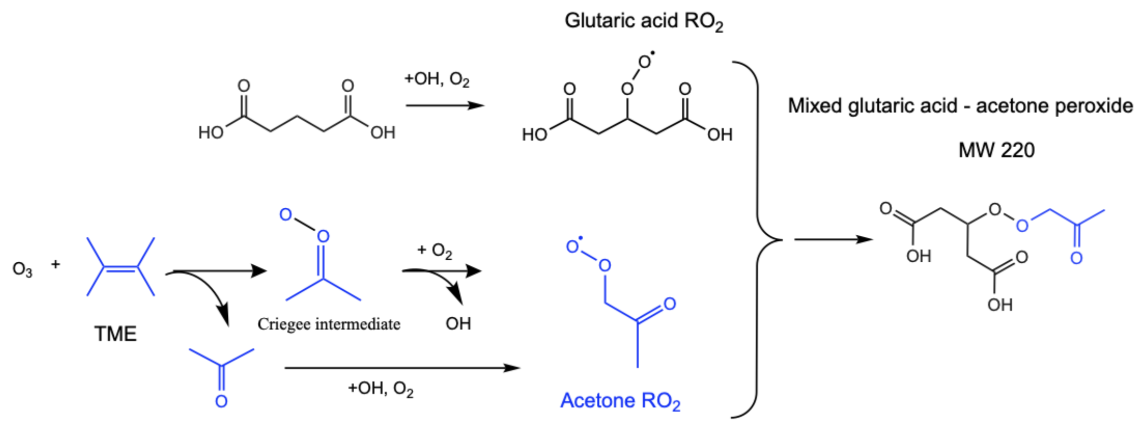

Text S3:

Product identification was further carried out with MAIV (+) MS/MS at collision energies (CE) ranging from 5 to 20 eV in the positive ion mode (Fig. S2-S5). All products are ammoniated parent peaks, thus it is expected that they each lose neutral NH_3 first, leaving behind protonated molecules that are consistent with the molecular weights of the products.[10] For the $[M + NH_4]^+$ adduct at *m/z* 164 attributed to the ketone product, the following fragmentations include loss of H_2O (*m/z* 129) and then either H_2O (*m/z* 111) or CO (*m/z* 101) and finally either CH_2CO (*m/z* 59) or HCOOH (*m/z* 55) at CE = 20 eV. For the $[M + NH_4]^+$ adduct at *m/z* 166 attributed to the alcohol product, the fragmentations include loss of H_2O (*m/z* 131), likely from the alcohol group first, then loss of H_2O (*m/z* 113) and CO (*m/z* 85), as would be expected for an acid group. For the $[M + NH_4]^+$ adduct at *m/z* 182 attributed to the hydroperoxide, the fragmentations include loss of one H_2O molecule (*m/z* 147, RO^+) and further loss of a CH_3COOH (*m/z* 87) and then CO_2 (*m/z* 43). Finally, for the $[M + NH_4]^+$ adduct at *m/z* 312 attributed to the ROOR, the

fragmentation begins with the loss of two H_2O molecules (*m/z* 277 and *m/z* 259), most likely from two of the four acid groups, and further loss of CO (*m/z* 231). At higher collision energy, fragmentation occurs at the O-O bond, forming an RO^+ fragment (*m/z* 147), followed by losses of O (*m/z* 131), H_2O (*m/z* 113), and CO (*m/z* 85), or another route by losing O (*m/z* 131), CO_2 (*m/z* 87), and CO_2 (*m/z* 43), consistent with a peroxide.

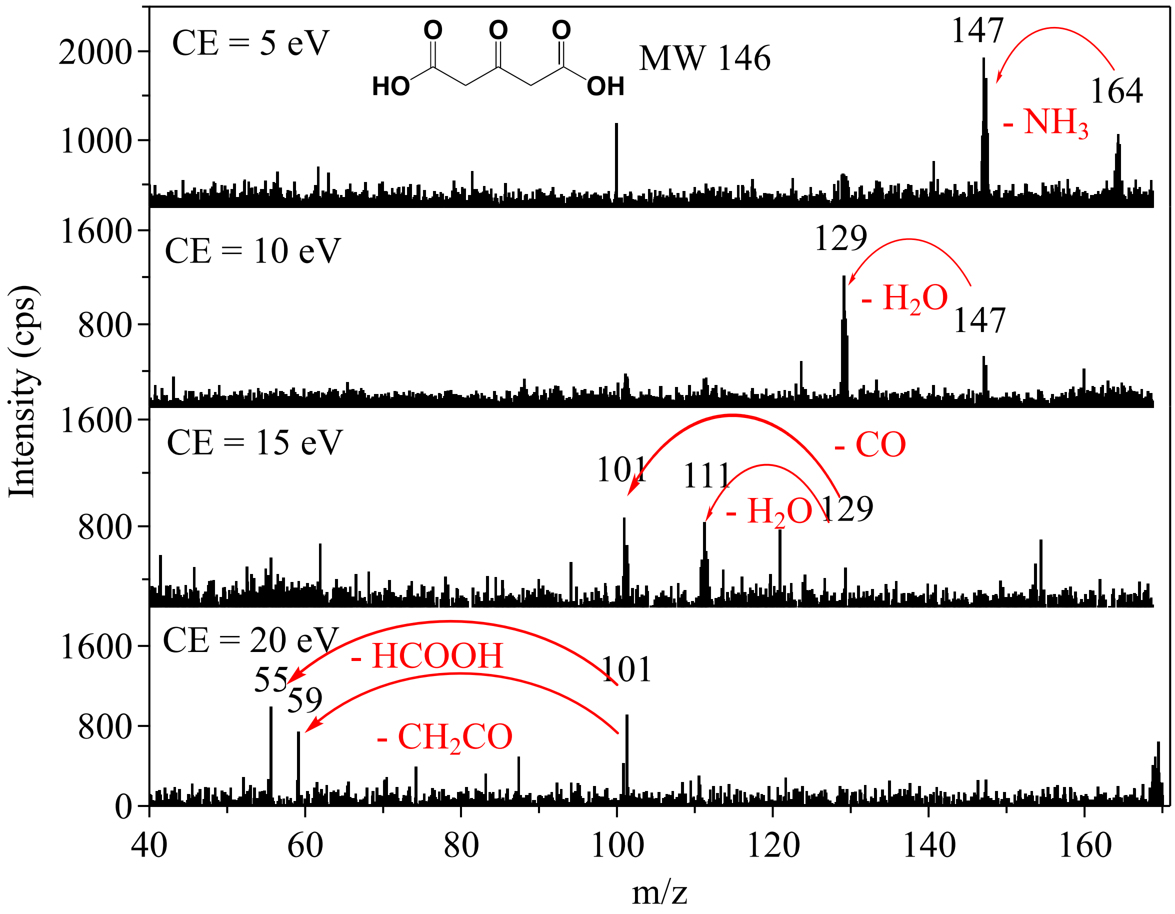

Figure S2. MAIV (+) MS/MS spectra showing the fragmentation pattern of the product at *m/z* 164 at collision energies (CE) from 5 to 20 eV. The expected R=O product has a MW of 146.

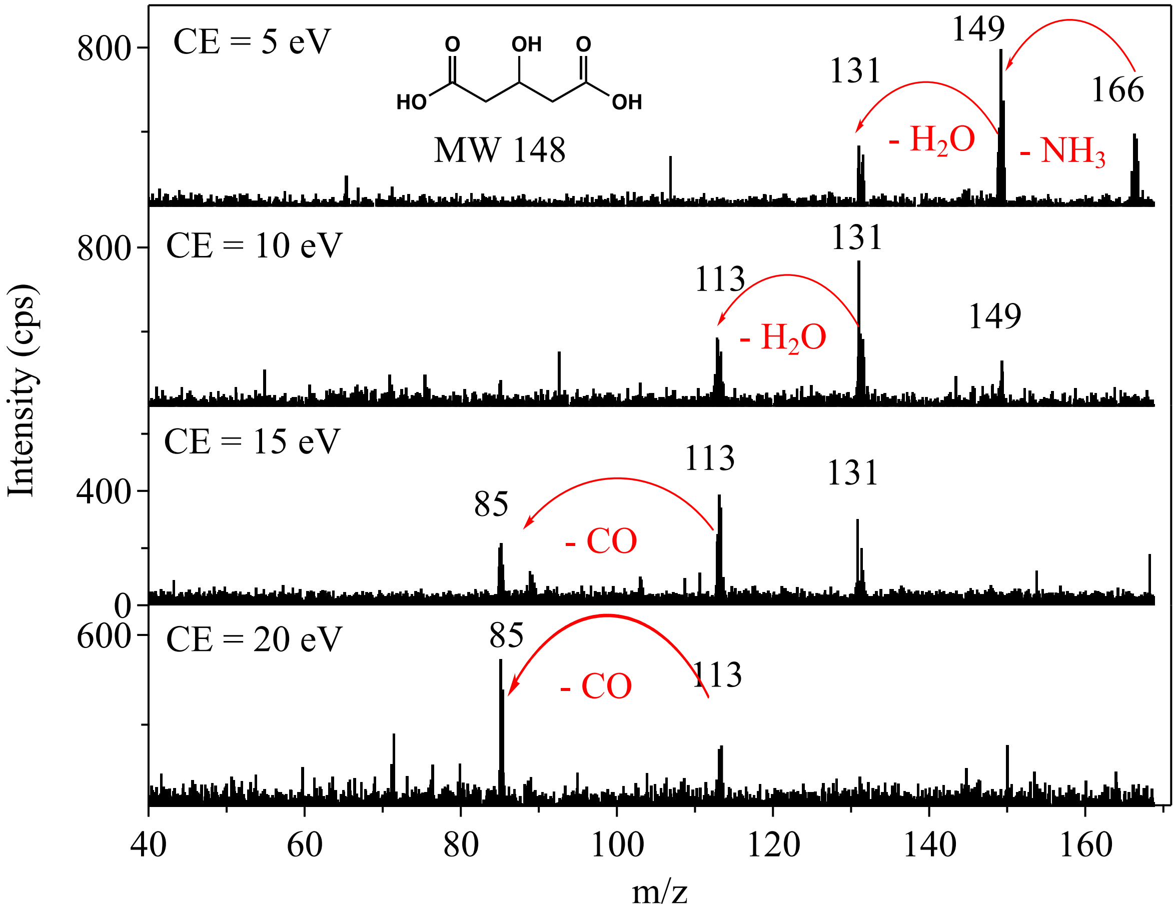

Figure S3. MAIV (+) MS/MS spectra showing the fragmentation pattern of the product at *m/z* 166 at collision energies (CE) from 5 to 20 eV. The expected ROH product has a MW of 148.

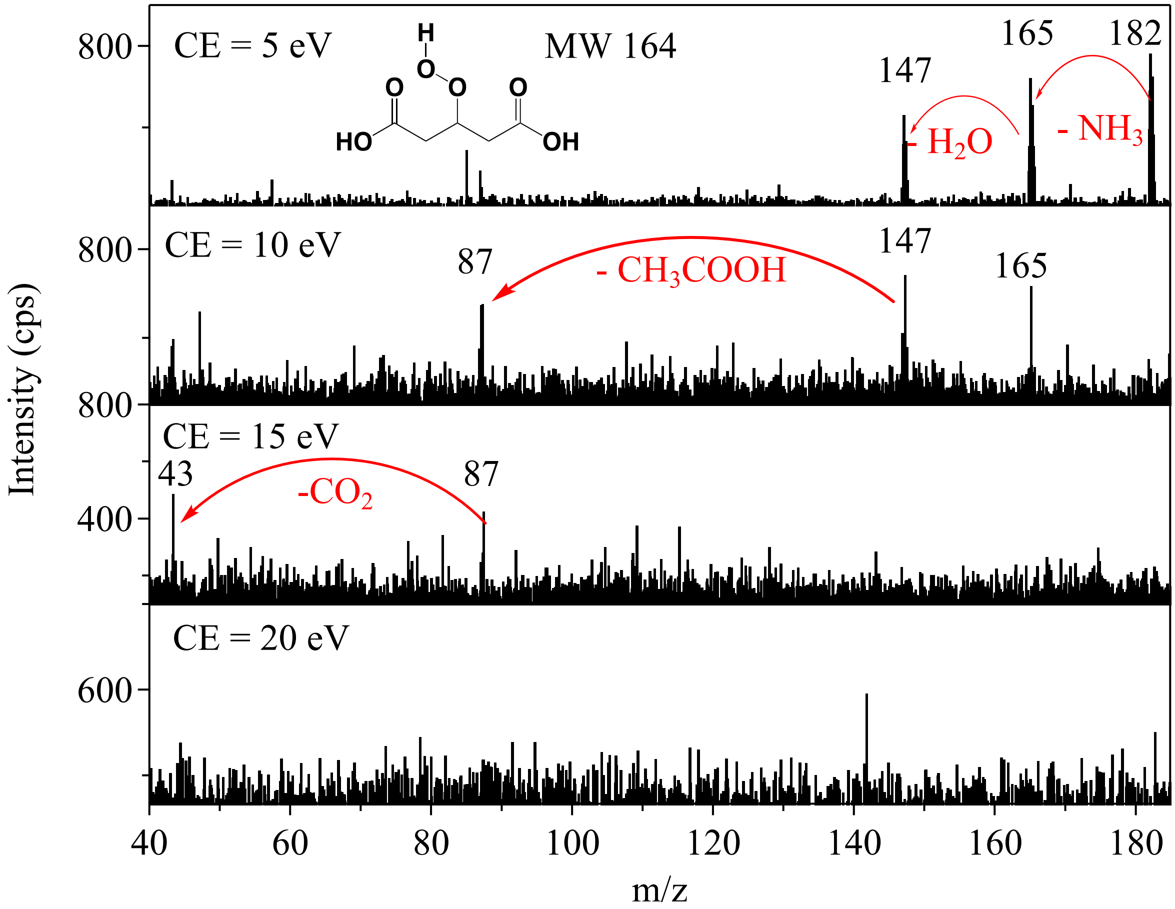

Figure S4. MAIV (+) MS/MS spectra showing the fragmentation pattern of the product at *m/z* 182 at collision energies (CE) from 5 to 20 eV. The expected ROOH product has a MW of 164.

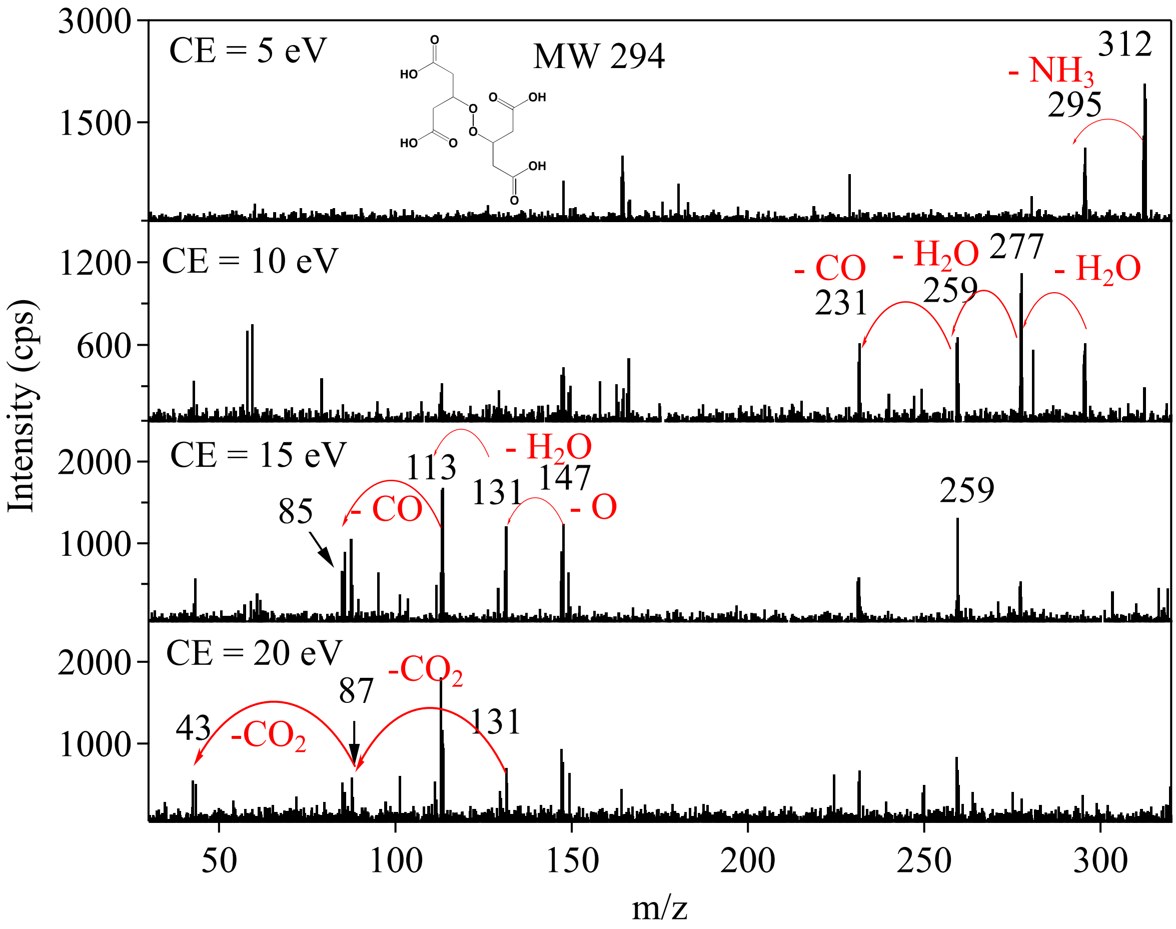

Figure S5. MAIV (+) MS/MS spectra showing the fragmentation pattern of the product at *m/z* 312 at collision energies (CE) from 5 to 20 eV. The expected ROOR product has a MW of 294.

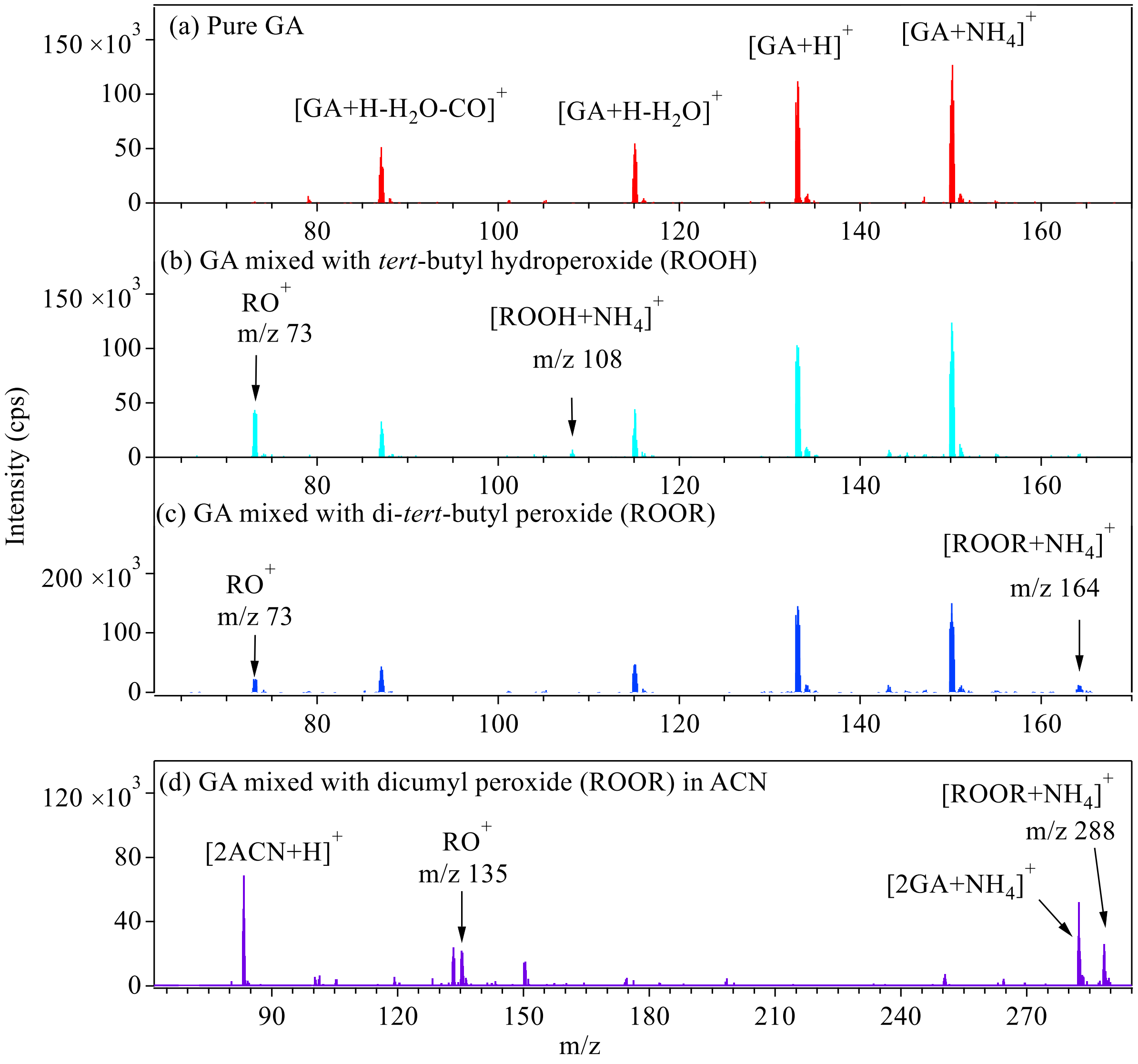

Figure S6. MAIV (+) mass spectra of organic peroxide and organic hydroperoxide commercial standards mixed with glutaric acid. Note that the source temperature was kept as 30°C for these experiments. The dicumyl peroxide:GA solution was prepared in acetonitrile due to the water insolubility of the peroxide: (a) Pure glutaric acid particles for reference. The molar ratios of glutaric acid to the standards in solution were (b) 1:1.7 for glutaric acid to *tert*-butyl hydroperoxide, (c) 1:0.7 for glutaric acid to di-*tert*-butyl peroxide, and (d) 1:0.3 for glutaric acid to dicumyl peroxide. The molar ratio and the distribution of the two solutes in the particle phase

could not be measured directly and may not be the same as the solutions from which they were formed.

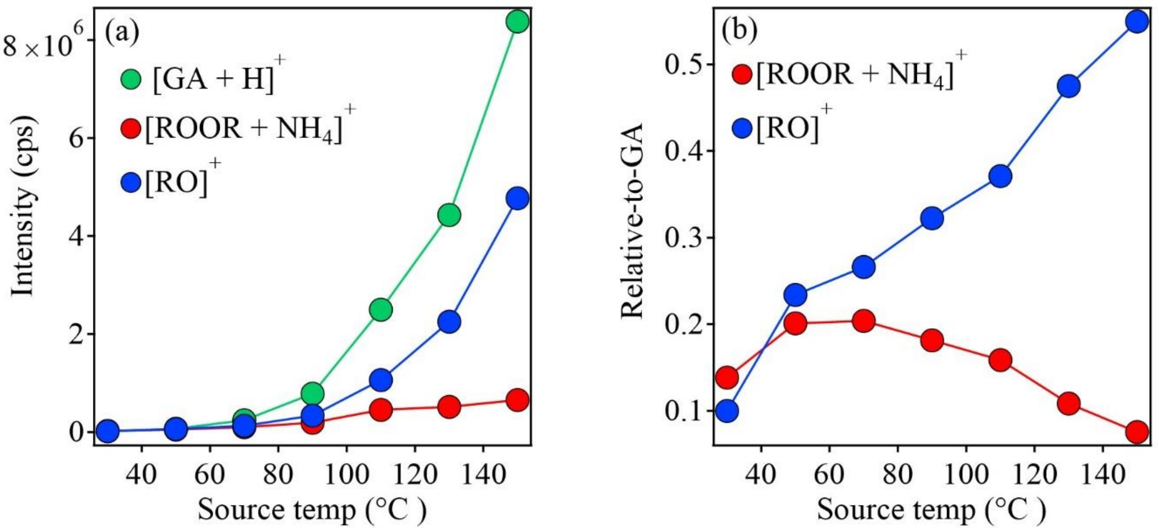

Figure S7. Temperature dependence of the MAIV (+) signal intensities of glutaric acid and ROOR for the particles atomized from the 1:0.3 glutaric acid to dicumyl peroxide solution. The x-axis is the source temperature on the mass spectrometer whereas the particles experience a lower temperature as they pass through the inlet. The 150°C source temperature corresponded to 61°C from measurement while 30°C corresponded to 28°C. (a) Signal intensities, and (b) intensities relative to GA.

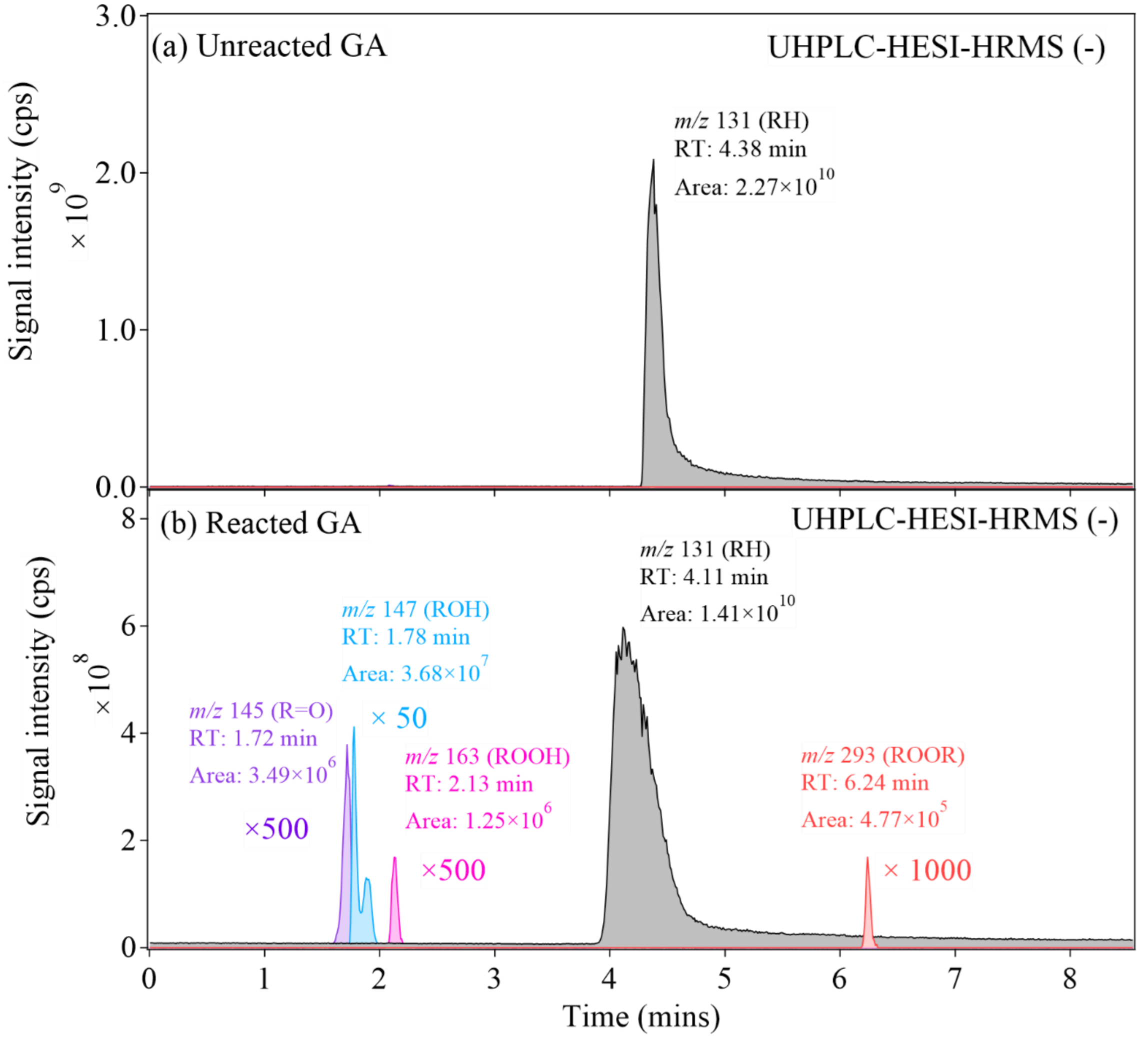

Figure S8. UHPLC-HESI-HRMS (-) extracted ion chromatograms (EIC) of (a) unreacted GA particles at *m/z* 131 $[GA - H]^-$, and (b) reactant GA and its oxidation products at *m/z* 145 $[(R{=}O) - H]^-$, *m/z* 147 $[ROH - H]^-$, *m/z* 163 $[ROOH - H]^-$, *m/z* 293 $[ROOR - H]^-$. The product signals are scaled for visualization in (b).

Table S1: Retention times, accurate masses, exact masses, assigned formulae, mass accuracy, and peak area for each product observed using UHPLC-HESI-HRMS (-).

**Compound***	**RT (min)**	**Accurate mass [M - H]$^-$**	**Exact mass [M - H]$^-$**	**Formula**	**Delta (ppm)**	**Area**
GA<br>Glutaric acid	4.11	131.0348	131.0349	$C_5H_7O_4^-$	-0.76	1.41×10^{10}
R=O<br>Ketone	1.72	145.0143	145.0142	$C_5H_5O_5^-$	0.04	3.49×10^{6}
ROH<br>Alcohol	1.78	147.0299	147.0299	$C_5H_7O_5^-$	-0.03	3.68×10^{7}
ROOH<br>Hydroperoxide	2.13	163.0249	163.0248	$C_5H_7O_6^-$	0.56	1.25×10^{6}
ROOR<br>Peroxide	6.24	293.0516	293.0514	$C_{10}H_{13}O_{10}^-$	0.63	4.77×10^{5}

*Products include all isomers

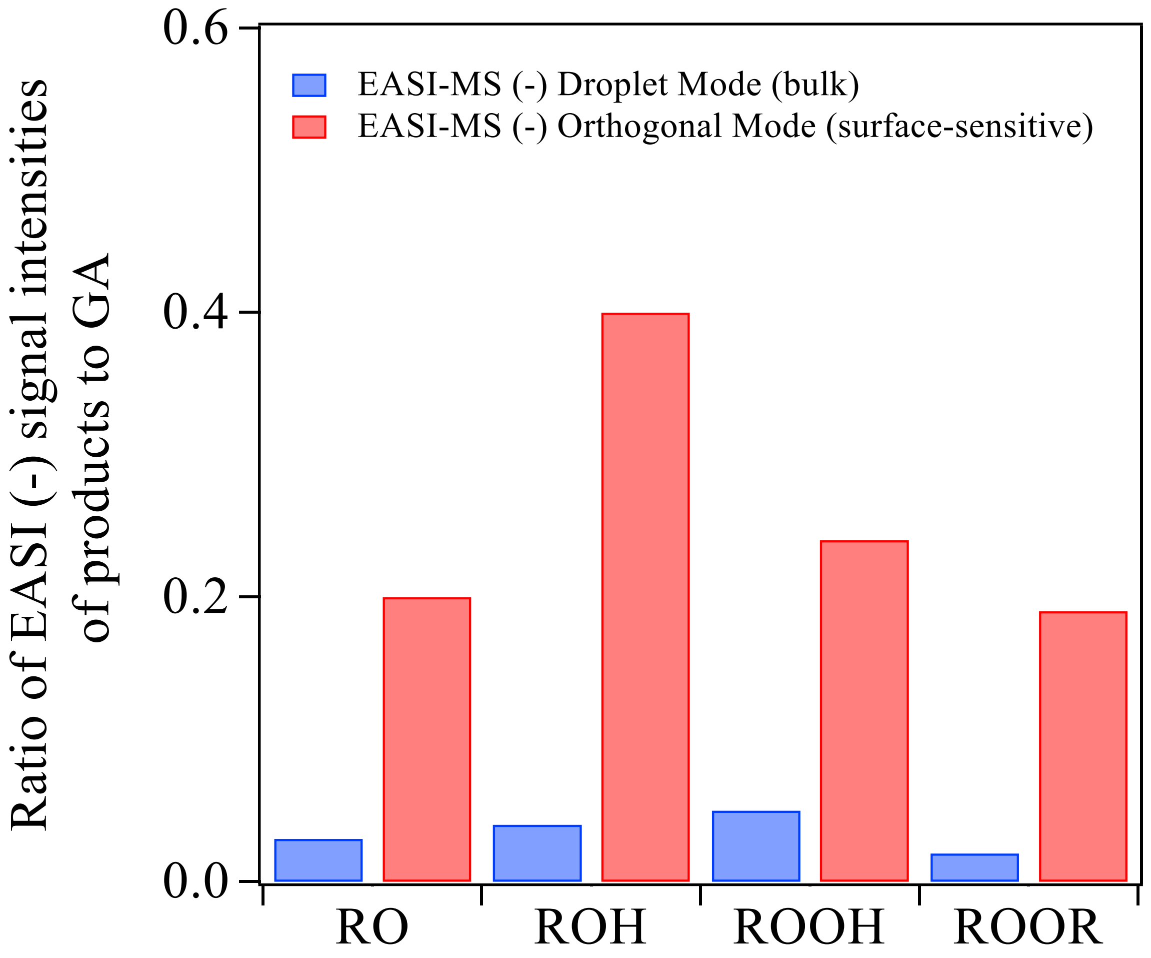

Figure S9. Ratios of product signal intensities relative to glutaric acid signal for EASI-MS (-) droplet mode (blue) and orthogonal mode (red).

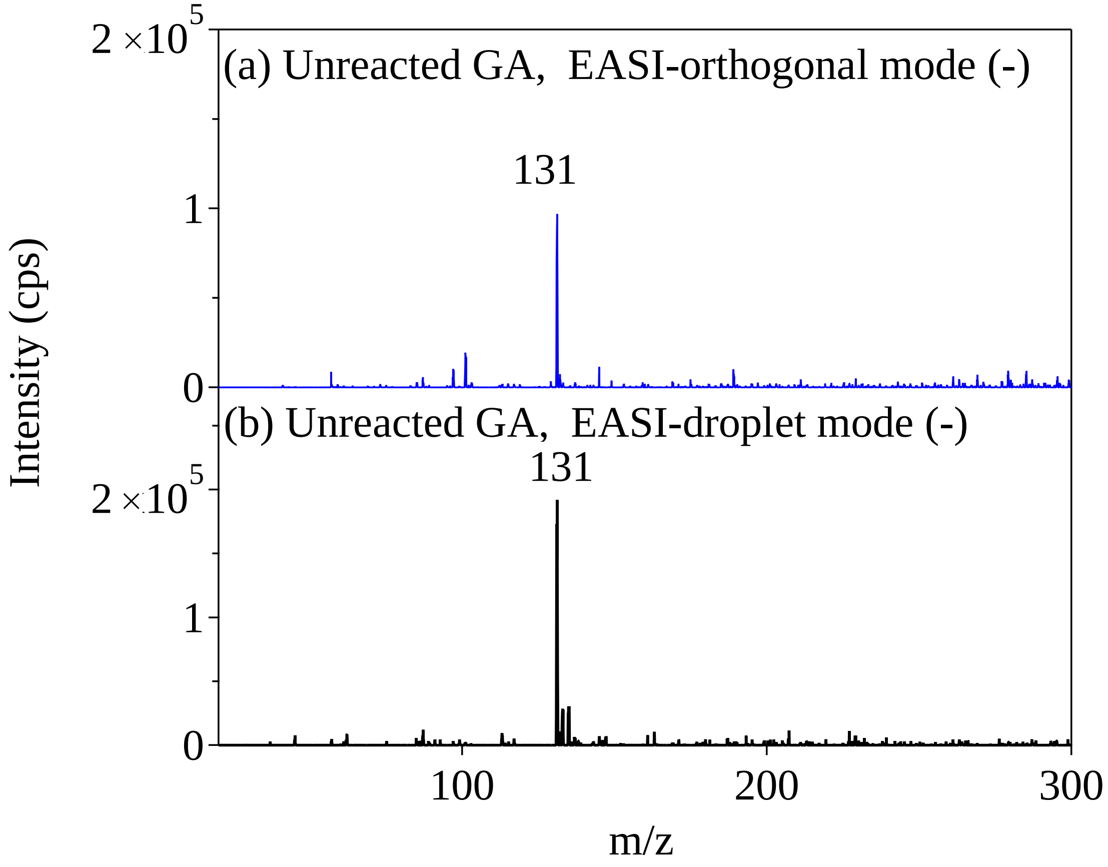

Figure S10. EASI (-) mass spectra in the EASI-orthogonal mode and the EASI-droplet mode for unreacted glutaric acid particles.

References:

(1) Houle, F. A.; Wiegel, A. A.; Wilson, K. R. Changes in Reactivity as Chemistry Becomes Confined to an Interface. the Case of Free Radical Oxidation of $C_{30}H_{62}$ Alkane by OH. *J. Phys. Chem. Lett.* **2018**, *9* (5), 1053–1057. https://doi.org/10.1021/acs.jpclett.8b00172.

(2) Eliason, T. L.; Gilman, J. B.; Vaida, V. Oxidation of Organic Films Relevant to Atmospheric Aerosols. *Atmos. Environ.* **2004**, *38* (9), 1367–1378. https://doi.org/10.1016/j.atmosenv.2003.11.025.

(3) Knopf, D. A.; Mak, J.; Gross, S.; Bertram, A. K. Does Atmospheric Processing of Saturated Hydrocarbon Surfaces by NO_3 Lead to Volatilization? *Geophys. Res. Lett.* **2006**, *33* (17), 1–5. https://doi.org/10.1029/2006GL026884.

(4) Docherty, K. S.; Ziemann, P. J. Reaction of Oleic Acid Particles with NO_3 Radicals: Products, Mechanism, and Implications for Radical-Initiated Organic Aerosol Oxidation. *J. Phys. Chem. A* **2006**, *110* (10), 3567–3577. https://doi.org/10.1021/jp0582383.

(5) Molina, M. J.; Ivanov, A. V.; Trakhtenberg, S.; Molina, L. T. Atmospheric Evolution of Organic Aerosol. *Geophys. Res. Lett.* **2004**, *31* (22), 1–5. https://doi.org/10.1029/2004GL020910.

(6) Vlasenko, A.; George, I. J.; Abbatt, J. P. D. Formation of Volatile Organic Compounds in the Heterogeneous Oxidation of Condensed-Phase Organic Films by Gas-Phase OH. *J. Phys. Chem. A* **2008**, *112* (7), 1552–1560. https://doi.org/10.1021/jp0772979.

(7) Chemspider. CSID:845. http://www.chemspider.com/Chemical-Structure.845.html.

(8) Bilde, M.; Barsanti, K.; Booth, M.; Cappa, C. D.; Donahue, N. M.; Emanuelsson, E. U.; McFiggans, G.; Krieger, U. K.; Marcolli, C.; Topping, D.; Ziemann, P.; Barley, M.;

Clegg, S.; Dennis-Smither, B.; Hallquist, M.; Hallquist, Å. M.; Khlystov, A.; Kulmala, M.; Mogensen, D.; Percival, C. J.; Pope, F.; Reid, J. P.; Ribeiro Da Silva, M. A. V.; Rosenoern, T.; Salo, K.; Soonsin, V. P.; Yli-Juuti, T.; Prisle, N. L.; Pagels, J.; Rarey, J.; Zardini, A. A.; Riipinen, I. Saturation Vapor Pressures and Transition Enthalpies of Low-Volatility Organic Molecules of Atmospheric Relevance: From Dicarboxylic Acids to Complex Mixtures. *Chem. Rev.* **2015**, *115* (10), 4115–4156. https://doi.org/10.1021/cr5005502.

(9) Berndt, T.; Scholz, W.; Mentler, B.; Fischer, L.; Herrmann, H.; Kulmala, M.; Hansel, A. Accretion Product Formation from Self- and Cross-Reactions of RO_2 Radicals in the Atmosphere. *Angew. Chemie - Int. Ed.* **2018**, *57* (14), 3820–3824. https://doi.org/10.1002/anie.201710989.

(10) Greaves, J.; Roboz, J. *Mass Spectrometry for the Novice*; 2008; Vol. 319. https://doi.org/10.1126/science.319.5866.1115.
